# Functional Interactions between BK_Ca_ *α*-Subunit and Annexin A5: Implications in Apoptosis

**DOI:** 10.1155/2016/1607092

**Published:** 2016-09-25

**Authors:** Stephen P. Brazier, Vsevolod Telezhkin, Paul J. Kemp

**Affiliations:** Division of Pathophysiology and Repair, School of Biosciences, Sir Martin Evans Building, Museum Avenue, Cardiff University, Cardiff CF10 3AX, UK

## Abstract

Proteomic studies have suggested a biochemical interaction between *α* subunit of the large conductance, voltage- and Ca^2+^-activated potassium channel (BK_Ca_ *α*), and annexin A5 (ANXA5), which we verify here by coimmunoprecipitation and double labelling immunocytochemistry. The observation that annexin is flipped to the outer membrane leaflet of the plasma membrane during apoptosis, together with the knowledge that the intracellular C-terminal of BK_Ca_ *α* contains both Ca^2+^-binding and a putative annexin-binding motif, prompted us to investigate the functional consequences of this protein partnership to cell death. Membrane biotinylation demonstrated that ANXA5 was flipped to the outer membrane leaflet of HEK 293 cells early in serum deprivation-evoked apoptosis. As expected, serum deprivation caused caspase-3/7 activation and this was accentuated in BK_Ca_ *α* expressing HEK 293 cells. The functional consequences of ANXA5 partnership with BK_Ca_ *α* were striking, with ANXA5 knockdown causing an increase and ANXA5 overexpression causing a decrease, in single BK_Ca_ channel Ca^2+^-sensitivity, measured in inside-out membrane patches by patch-clamp. Taken together, these data suggest a novel model of the early stages of apoptosis where membrane flippage results in removal of the inhibitory effect of ANXA5 on K^+^ channel activity with the consequent amplification of Ca^2+^ influx and augmented activation of caspases.

## 1. Introduction

Programmed cell death (apoptosis) is a series of controlled events which, when balanced with cell proliferation, is essential for the normal development of tissue and function of cells. Disturbing this equilibrium is a contributory factor in a number of disease states, such as neurodegenerative disorders, pulmonary hypertension, and cancer [1–3]. Apoptosis occurs in three main steps: (i) the initiation event when the signal for a cell to apoptosis is received and the cell begins to shrink, due to loss of cell volume; (ii) the effector phase when the mitochondrial membrane potential becomes depolarized, the mitochondrial transition pore opens, and the release of cytochrome C and other large molecules from the mitochondrial matrix results in activation of caspases; and (iii) the last phase in which DNA becomes degraded, apoptotic bodies excise from the plasma membrane, and the cytoskeleton is broken down (see [4] for review). As part of this process, phosphatidylserine is translocated from the inner (cytoplasmic) leaflet of the plasma membrane to the outer (cell surface) leaflet soon after the induction of apoptosis (“flippage”), where proteins such as the annexins are known to bind with high affinity [5]. This flippage phenomenon is utilized in diagnostic tests for apoptosis. However, little evidence exists as to how and when flippage occurs and what are the consequences that this process has for the apoptosis cascade.

K^+^ fluxes have been shown to play an important role in both the early and late phases of apoptosis [4]. Once apoptosis is triggered, one of the earliest observed morphological changes is cell shrinkage or apoptotic volume decrease (AVD). This is due to the efflux of K^+^ and Cl^−^ ions through their respective channels that leads to water exiting the cell through aquaporin water channels in order to maintain the osmotic pressure balance between internal and external compartments. Identification of the specific K^+^ channels, which are involved in apoptotic volume decrease, has employed pharmacological agents; to date, evidence suggests that all the major classes of K^+^ channels (voltage-gated, Ca^2+^-activated, ATP-sensitive, inwardly rectifying, and two pore domain K^+^ channels) have some role to play in apoptosis [8–12]. Blockage of the large conductance and voltage- and calcium-activated potassium channel (BK_Ca_) by iberiotoxin or tetraethylammonium (TEA) inhibits carbonyl cyanide 4-(trifluoromethoxy)phenylhydrazone (FCCP, a mitochondrial protonophore) induced apoptosis in both rat and human pulmonary smooth muscle cells [13]. Other studies indicate that the application of NS1619, a specific BK_Ca_ channel opener which can induce apoptosis in ovarian cancer cells [14] and exposure of erythrocytes to ionomycin (Ca^2+^ ionophore which raises internal Ca^2+^ concentrations ([Ca^2+^]_i_)) induces cell shrinkage and apoptosis via BK_Ca_ [15]. During the later stages of apoptosis, it has been demonstrated that only cells with a decreased cytosolic concentration of K^+^ exhibit caspase activity [16, 17], suggesting that the efflux of K^+^ during AVD is permissive for caspase activation during the later stages of cell death.

The annexins are a structurally related family of Ca^2+^-sensitive proteins which participate in a wide range of cellular functions, including cellular signalling, cell migration, proliferation, and apoptosis [18–21]. These proteins have been shown to interact with a number of ion channels and are important in both membrane trafficking and modifying of ion channel function [1, 22, 23]. Using a proteomics-based approach, which previously identified heme oxygenase-2 (HO-2) as O_2_-sensing protein partner of BK_Ca_, we also identified ANXA5 as a potential interacting protein of BK_Ca_ [24]. A more recent study has shown that this particular protein partnership also occurs in native tissues, namely, the mouse cochlea [25]. Based on the knowledge that BK_Ca_ channel activation can induce apoptosis in a number of different cell types and the fact that annexins bind to externalized phosphatidylserine in response to apoptosis, we have begun to investigate how the partnership between ANXA5 and BK_Ca_ might influence this process. It has been proposed that annexins have a binding affinity for EF-hand Ca^2+^-binding proteins and *α*-subunit of the human BK_Ca_ (KCNMA1) has a similar domain within its Ca^2+^ sensing region [26].

The aim of this study was to validate the interaction of ANXA5 and BK_Ca_ *α*-subunit (BK_Ca_ *α*), which was previously suggested by proteomics [24], to determine the biophysical consequences of the BK_Ca_ *α*/ANXA5 partnership, in particular to elucidate the structural elements required, and to define the impact of this partnership on apoptosis.

## 2. Materials and Methods

### 2.1. Cell Culture

The studies were performed on both wild type HEK 293 cells and HEK 293 cell line stably expressing the human BK_Ca_ *α* subunit (see [24, 27–31]). HEK 293 cells were maintained in Earle's minimal essential medium (containing L-glutamine) supplemented with 10% fetal calf serum, 1% antibiotic/antimycotic, and 1% nonessential amino acids (Gibco BRL, Strathclyde, UK) in a humidified incubator gassed with 5% CO_2_/95% air. Where indicated, apoptosis was induced by 48 h of serum deprivation (SD). Knockdown and overexpression of ANXA5 were achieved by transient transfection of cells with specific siRNA and ANXA5 plasmid DNA, respectively. Transfection was achieved using the Amaxa Nucleofector Kit V supplied with green fluorescent protein (GFP) for positive identification of transfected cells, following the manufacturer's protocols (Amaxa Biosystems, Germany). 24 h before performing the electrophysiological experiments, cells were passaged and plated onto glass coverslips, cultured as above, and then transferred into a continuously perfused (5 mL·min^−1^) recording chamber (volume* ca.* 200 *μ*L) mounted on the stage of an inverted microscope equipped with phase-contrast and fluorescent optics for (Olympus CK40).

### 2.2. Electrophysiological Recordings

BK_Ca_ *α* currents were recorded from inside-out membrane patches excised from the stably expressing the wild type HEK293 cells, transiently transfected with the ANXA5 siRNA or the ANXA5 plasmid. The pipette and bath solutions contained (in mM) 10 NaCl, 117 KCl, 2 MgCl_2_, 11 N-2-hydroxyethylpiperazine-N′-2-ethanesulfonic acid (HEPES; pH 7.2) with [Ca^2+^]_o_ at 1.2 mM and [Ca^2+^]_i_ adjusted to the quasiphysiological level of 300 nM using ethylene-glycol-tetra-acetic acid (EGTA), except when experiments were performed at the different range of Ca^2+^ concentration (10 nM–1 mM). The CO donor molecule, tricarbonyldichlororuthenium (II) dimer ([Ru(CO_3_)Cl_2_]_2_, Sigma-Aldrich, Poole, Dorset, UK) (CORM-2), was employed to release free CO in the experimental solution [32].

All recordings were performed at the room temperature (22 ± 0.5°C) using an Axopatch 200 A amplifier and Digidata 1320 A/D interface (Axon Instruments, Forster City, CA, USA). BK_Ca_ *α* channel macroscopic currents were recorded using a protocol of 8 s voltage ramp from −30 mV to 120 mV, followed by three, 250 ms steps to +40, +60, and +120 mV, repeated at 0.1 Hz. All voltages are reported with the respect to the inner membrane leaflet (–Vp). All recordings were filtered with 8-pole Bessel filter at 5 kHz and digitized at 10 kHz. Half-activation voltages (Va_50_) and half-activation concentrations (EC_50_) were obtained from the current-voltage and concentration-response curves, respectively, and were fitted using Hill equation. Statistical comparisons were performed using one-way ANOVA and the differences were considered significant at the level of *p* < 0.05.

### 2.3. Caspase Assays

Apoptosis studies were performed using the Caspase-Glo 3/7 assay system (Promega). HEK 293 BK_Ca_ Cells and HEK 293 BK_Ca_ cells transfected with ANXA5 siRNA (see above) were plated into 96-well white walled plates, in triplicate for each experimental condition. The following day apoptosis was induced by replacing the media with DMEM supplemented with 8% mannitol (serum-withdrawal media). Cells were then analysed for caspase-3/7 activity at 24-hour and 48-hour time points. Briefly, 100 *μ*L of Caspase-Glo 3/7 reagent was added to each well and the plate was shaken at 300 rpm for 30 seconds; the plates were then incubated at room temperature for one hour before being assayed in a plate reading luminometer (Fluroskan Ascent FL, Thermo Labsystems). Duplicate plates were seeded at the start of all our apoptosis experiments; this allowed us to normalize our luminescence readings to cell count number. Cell count number was determined by using CyQUANT cell proliferation assay (Invitrogen), following the manufacturers' instructions.

### 2.4. Reciprocal Coimmunoprecipitations

Wild type HEK 293 cells were seeded into T75 flask and grown to about 80% confluency. The cells were then washed twice with ice cold PBS before adding 1 mL of ice cold RIPA buffer containing protease inhibitor cocktail (Sigma) to the cells. The cells were then scrapped from the flask and transferred to a precooled centrifuge tube and placed on a rocker for 30 minutes at 4°C. The lysate was then centrifuged at 14,000 ×g for 15 minutes and the supernatant transferred to a fresh tube. Protein G Dynabeads (Invitrogen) was prepared following the manufacturers' instructions, 50 *μ*L of prepared Dynabeads was resuspended in 100 *μ*L citrate phosphate buffer (pH 5), and either 5 *μ*g of BK_Ca_ *α* (Alamone) or ANXA5 (Santa Cruz) antibody was added. The tubes were then incubated with rotation for 40 minutes at room temperature. Ig-Dynabeads complex was then washed 3 times in citrate phosphate buffer (pH 5) containing 0.01% Tween-20 using a magnetic stand to capture the beads. After the final wash precleared lysate (protein samples incubated with prepared Dynabeads to reduce nonspecific binding) was added to Ig-Dynabeads complex and incubated with rotation for a further hour at room temperature. The beads were then washed 3 times in 1 mL of PBS and the protein complex eluted from the beads by adding 20 *μ*L 0.1 M citrate buffer (pH 2-3) and incubating for 2 minutes. Finally the pH of the eluate was adjusted by adding 1 M Tris pH 7.5. BK_Ca_ and ANXA5 were detected using standard western blotting techniques.

### 2.5. Immunocytochemistry

Recombinant HEK 293 BK_Ca_ *α* cells were plated onto coverslips for immunostaining. Media was aspirated from the dishes and cells were fixed with ice cold methanol for 10 minutes, followed by 3 washes with PBS. Cells were then blocked with 5% BSA for 1 hour at room temperature before applying the primary antibodies in 1% BSA. Both anti-BK_Ca_ *α* (Alamone) and anti-ANXA5 antibodies were used at 1 : 200 dilution. Antibodies were incubated overnight at 4°C with gently rocking followed by 3 washes with PBS. Following the final PBS wash appropriate TRITC and FITC conjugated secondary antibodies (Alexa) were added to the cells at a dilution of 1 : 500 and incubated at room temperature for 2 hours. The cells were washed 3 times with PBS and the coverslip was fixed onto a slide using VECTASHIELD (containing DAPI) and images taken with an Olympus BX61 camera (Olympus).

### 2.6. Surface Biotinylation and Western Blotting

HEK 293 BK_Ca_ *α* cells were seeded into T75 flasks and incubated for 48 hours in either normal or serum-withdrawal media. Pierces cell surface protein isolation kit (Thermo Scientific) was then used to biotinylate and extract cell surface membrane proteins. Cells were washed twice in ice cold PBS and 10 mL of biotin solution was added to each flask. The flasks were incubated at 4°C for 30 minutes on a rocking platform before terminating the reactions by adding 0.5 mL of quenching solution. Cells were gently scrapped into solution and pelleted by centrifugation before being resuspended in lysis solution. Cells were then incubated on ice for 30 minutes, vortexing every 5 minutes for 5 seconds. The cell lysate was then centrifuged at 10,000 ×g for 2 minutes and the biotin labelled proteins were isolated from the supernatant using a NeutrAvidin agarose column. Protein samples were separated by electrophoresis on 10% acrylamide gels and annexin A5 was detected by western blotting using an anti-ANXA5 antibody purchased from Santa Cruz antibodies.

## 3. Results

### 3.1. Interaction and Colocalization of BK_Ca_ *α* with ANXA5

**Figure 1:**
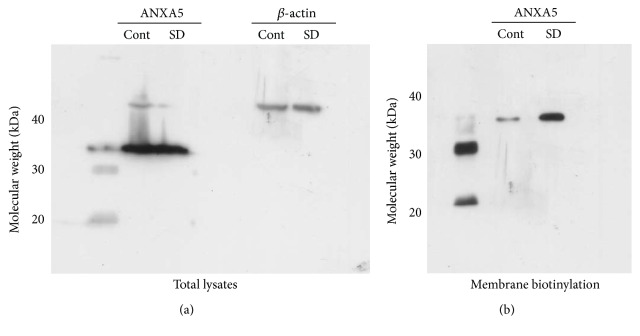
Serum deprivation-evoked apoptosis results in early localization of ANXA5 to plasma membrane outer leaflet. (a) The total cell lysates of BK_Ca_ *α* HEK239 cells cultured for 48 h in either normal (Cont) or serum-deprived (SD) media where prepared and western blotted to quantify the amounts of ANXA5 and *β*-actin. (b) HEK 293 cells stably expressing the human BK_Ca_ *α* subunit where incubated for 48 hours in before being incubated with biotin reagent. Extracts where prepared and normalized for protein content before the cell surface proteins were isolated with avidin-agarose beads. ANXA5 was detected on the blot using a specific antibody.

**Figure 2:**
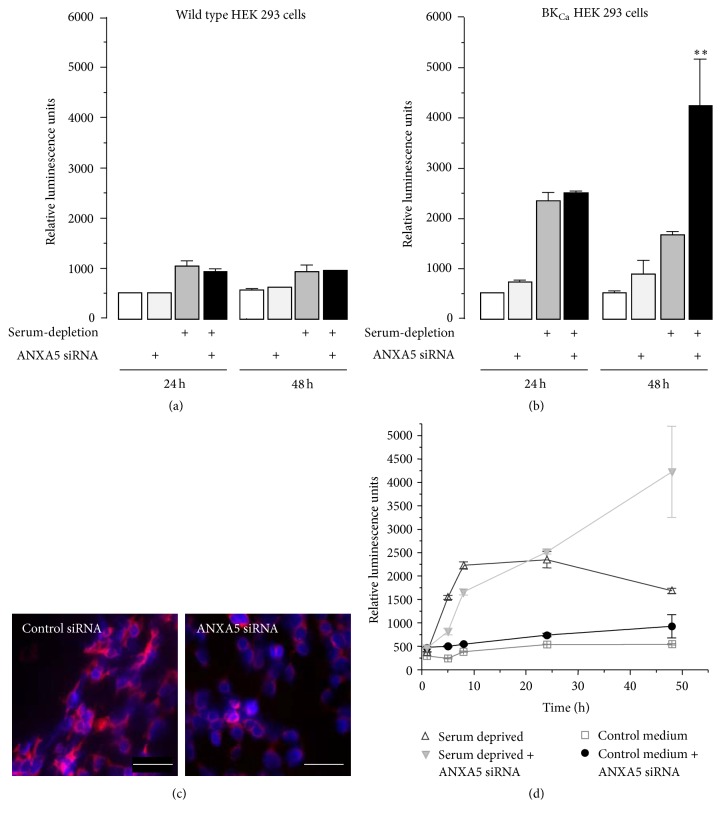
BK_Ca_ *α* augments the apoptotic response to serum deprivation: a response which is amplified by ANXA5 knockdown. (a) Caspase-3/7 activity in wild type HEK 293 cells was quantified as relative luminescence units at 24 h and 48 h. Apoptosis was induced by serum deprivation at time = 0 h, with and without ANXA5 knockdown using siRNA (shown beneath the bars). (b) As (a), but using BK_Ca_ *α* HEK239 cells. Asterisks indicate that, after 48 h, ANXA5 knockdown enhanced the serum-deprived-evoked caspase activation (*p* < 0.05, *n* = 3). (c) Immunocytochemistry using an antibody directed against ANXA5 in BK_Ca_ *α* expressing HEK 293 cells following treatment with control (left) and ANXA5 (right) siRNA. (d) Time course of caspase-3/7 activity, BK_Ca_ *α* HEK 293 cells (± ANXA5 knockdown) incubated in the presence of normal medium (control), or serum-deprived media (8% Mannitol, DMEM), over a 48-hour period.

Although binding of ANXA5 to phosphatidylserine that results in its exposure to the external leaflet of the plasma membrane is routinely used as an assay of cell death, little information is available on the potential role of annexins in the earlier stages of apoptosis. The effect that serum deprivation had on ANXA5 protein levels within the cell was elucidated using western blotting (Figure 1). Western blots of total lysates of cells incubated for 48 h in either serum-deprived (SD) or normal medium showed no difference in total ANXA5 expression levels (Figure 1(a)). However, western blot of extracts of cells, which had been surface biotinylated, demonstrated that serum deprivation resulted in a dramatic rise in the amount of ANXA5 at the outer membrane leaflet. These data evidently demonstrate that significantly increased proportion of ANXA5 becomes exposed to the outer side of the plasma membrane during the initial stages of apoptosis. To test directly the extent to which ANXA5 and BK_Ca_ *α* channels contribute to the early stages of apoptosis, caspase-3/7 assays were performed at 24 and 48 h following serum deprivation (Figure 2). Within 24 h serum deprivation resulted in a significantly larger increase of caspase-3/7 activation in BK_Ca_ *α* HEK 293 cells than it did in the wild type HEK 293 cells; this difference was maintained up to the 48 h time point. This initial observation provided a decent evidence that BK_Ca_ *α* contributed to the early apoptotic response of HEK 293 cells. In the absence of serum deprivation the effect of ANXA5 siRNA knockdown demonstrated by immunocytochemistry (Figure 2(c)) was to increase significantly the caspase-3/7 activity in BK_Ca_ *α* HEK 293 cells only (Figure 2(b)). Importantly, when cells were deprived of serum, ANXA5 knockdown did not affect further the caspase-3/7 activation of either cell type at 24 h but induced a dramatic amplification of this apoptotic response at 48 h in BK_Ca_ *α* HEK 293 cells only (Figure 2(d)).

### 3.2. ANXA5 Modulates BK_Ca_ *α* Channel Function

**Figure 3:**
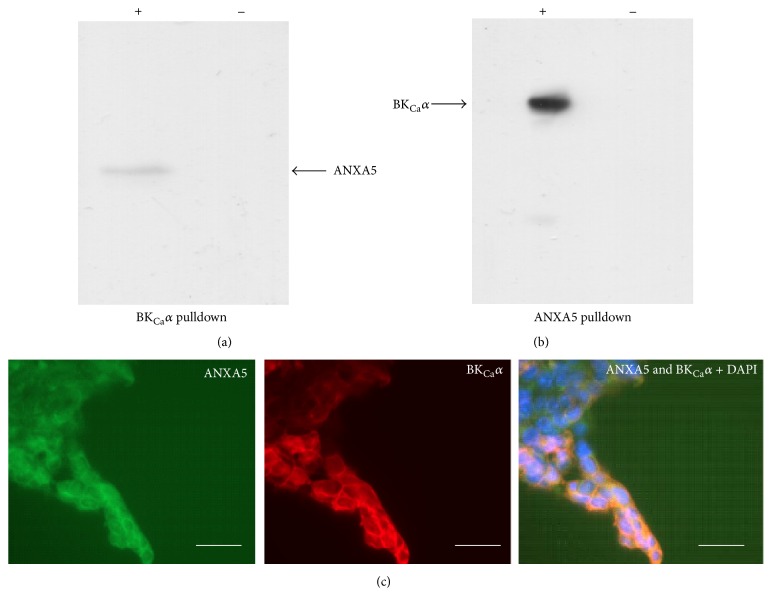
BK_Ca_ *α* subunit colocalizes with ANXA5. (a) Reciprocal coimmunoprecipitation from lysates of HEK 293 cells stably expressing BK_Ca_ *α* subunit with anti-BK_Ca_ *α* antibody (+). Bead controls (−) consisted of lysate mixed with DynaG beads lacking antibody. (b) Reciprocal coimmunoprecipitation from lysates of HEK 293 cells stably expressing BK_Ca_ *α* subunit with and anti-ANXA5 antibody (+) and bead control (−). (c) HEK 293 cells stably expressing BK_Ca_ *α* subunit were immunostained with anti-BK_Ca_ *α* subunit (1 : 250, red) and anti-ANXA5 (1 : 200 green) antibodies and cell nuclei (blue) where visualized with DAPI. Scale bars are 50 *μ*M.

A direct protein interaction between ANXA5 and BK_Ca_ *α* was validated using reciprocal coimmunoprecipitations of solubilized membrane proteins (Figure 3(a)) and immunocytochemistry (Figure 3(b)). The functional consequences of such physical interactions were then investigated using patch-clamp electrophysiology.

**Figure 4:**
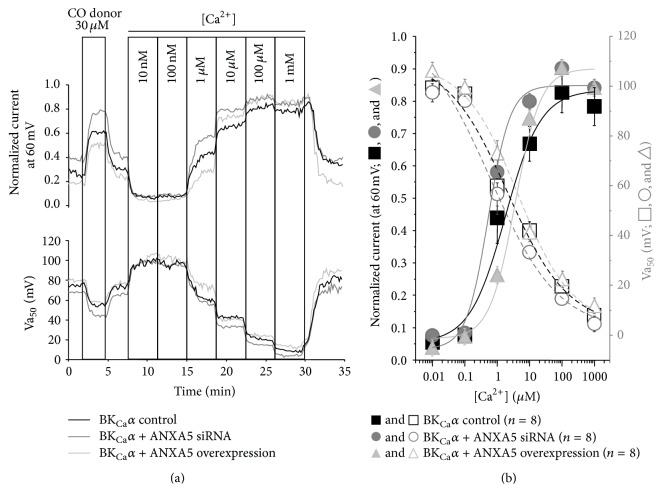
BK_Ca_ *α* channel function is modulated by its interaction with ANXA5 at different concentrations of Ca^2+^. (a) Time course showing the stimulatory effect of 30 *μ*M CORM-2 and the sequential increase of [Ca^2+^] in the concentration range 10 nM–1 mM on BK_Ca_ *α* subunit channel normalized current and Va_50_ in the control (black line), in the ANXA5 knockdown model achieved by transfection with siRNA (dark grey line), and in the ANXA5 overexpression model achieved by transfection with the DNA plasmid (light grey line). (b) Mean (± SEM) concentration-response plots displaying the stimulation effect of the sequential increase of [Ca^2+^] in the concentration range 10 nM–1 mM on BK_Ca_ *α* channel normalized current at the holding voltage of 60 mV in the wild type (black square and a solid black line, *n* = 8), in the ANXA5 knockdown model achieved by transfection with siRNA (grey circle and a solid grey line, *n* = 8), in the ANXA5 overexpression model achieved by transfection with ANXA5 DNA plasmid (light grey triangle and a solid light grey line, *n* = 8), on Va_50_ of BK_Ca_ *α* channels in the wild type (white square and a dashed black line, *n* = 8), in the ANXA5 knockdown model achieved by transfection with siRNA (white circle and a dashed grey line, *n* = 8), and in the ANXA5 overexpression model achieved by transfection with ANXA5 DNA plasmid (white triangle and a dashed light grey line, *n* = 8).

One of the most important physiological characteristics of BK_Ca_ channels is their ability to open in response to increase of [Ca^2+^]. Knockdown of ANXA5 using siRNA (confirmed by the immunocytochemistry shown in Figure 2(c)) resulted in a significant leftward shift of Ca^2+^ concentration-response curve of BK_Ca_ *α* channel activity (Figure 4). This was reflected in a significant change in the mean Va_50_ values (Figure 4 and Table 1), indicating increased Ca^2+^ sensitivity of BK_Ca_ *α*. Conversely, overexpression of ANXA5 (by transfection with a mammalian expression plasmid containing human ANXA5) evoked a significant rightward shift of Ca^2+^ concentration-response curve (Figure 4) and a change in Va_50_ values (Figure 4 and Table 1), indicating a decreased Ca^2+^ sensitivity. The alterations of Ca^2+^ sensitivities evoked by manipulation with ANXA5 expression occur within the physiological [Ca^2+^]_i_ window of between 100 nM and 1 *μ*M. Ca^2+^ sensitivities of BK_Ca_ *α* channels in the cells transfected with a scrambled siRNA or empty vector were not significantly different from those of the wild type HEK 293 cells (data are not illustrated).

The fact that sensitivity of BK_Ca_ *α* to Ca^2+^ was effectively modulated by ANXA5 expression was also approved by the observation that the control BK_Ca_ *α* activity, at the beginning of each trace in the presence of 300 nM [Ca^2+^]_i_, was higher or lower than normal. Independently of Ca^2+^ sensitivity relative activation of BK_Ca_ *α* by the carbon monoxide donor CORM-2 (30 *μ*M) was unaffected (Figure 4 and Table 2).

## 4. Discussion

**Figure 5:**
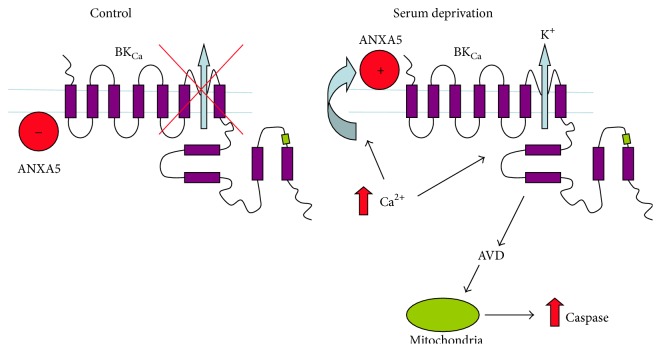
Proposed model to account for ANXA5/BK_Ca_ *α* interaction before and during apoptosis. During resting conditions, intracellular ANXA5 interacts with BK_Ca_ *α* and this partnership maintains the relatively low sensitivity to [Ca^2+^]_i_ of BK_Ca_ *α* channels. Thus, ANXA5 has an inhibitory effect on BK_Ca_ *α* channel activity and restricts K^+^ efflux. During serum deprivation, [Ca^2+^]_i_ increases and the channel begins to activate [6, 7] and ANXA5, which is associated with the phosphatidylserine, flips to the outer membrane leaflet, resulting in the physical uncoupling of BK_Ca_ *α*/ANXA5 interaction and a consequent increase in Ca^2+^ sensitivity. This augments channel activity further and results in an amplified K^+^ efflux and accelerates AVD, caspase activation, and cell death.

During the course of the lifetimes many cells of our body undergo programmed death and replacement by the new ones; this process is finely tuned by a variety of mechanisms within the body. However when this strictly controlled cell death/regeneration is impaired it can lead to the serious conditions such as AIDS and cancer. The earliest observable sign that a cell will undergo programmed cell death is cell shrinkage. This is primarily caused by K^+^ efflux through any of a steadily increasing number of K^+^ channels [8–12]. One of such K^+^ channels is BK_Ca_ of which activity is controlled by [Ca^2+^]_i_ that contributes to both maintenance of the resting membrane potential and repolarisation phase after action potential. Here we identify ANXA5 as a protein which physically communicates with BK_Ca_ *α*. The main aim of the present research was to understand how these two proteins interact at the functional level and to determine whether this interaction might have consequences for cell death. Previously it was considered that annexins have only been implicated in the last stage of apoptosis, but our data show that ANXA5 has a functional role in the earlier stages of apoptosis when caspases are activated, and this process is significantly enhanced in the presence of BK_Ca_ *α*. Based on this new information we propose that during resting conditions ANXA5 interacts with BK_Ca_ *α* on the intracellular side of the membrane; this protein partnership causes reduction of BK_Ca_ *α* channels sensitivity to [Ca^2+^]_i_; thus ANXA5 has an inhibitory effect on BK_Ca_ *α* channels' activity and keeps K^+^ efflux to the minimum. In contrast, when cells are exposed to the serum deprivation, two important processes are initiated. Firstly, [Ca^2+^]_i_ runs up and activates BK_Ca_ *α* [6, 7]. Secondly, ANXA5, which is associated with the phosphatidylserine, flips to the outer membrane leaflet, resulting in uncoupling of BK_Ca_ *α* /ANXA5 physical interaction and a consequent progressive increase of Ca^2+^ sensitivity of BK_Ca_ *α*. This augments BK_Ca_ *α* activity and results in an amplified K^+^ efflux which accelerates AVD, caspase activation, and cell death. This scheme is represented pictorially in Figure 5.

Although these experiments have been conducted in a heterologous expression system, they provide conception for how we view the control of apoptosis in a variety of tissues which express BK_Ca_ channels at a high level. For example, apoptosis is an important component of normal brain development and in the cerebellum such programmed cell death occurs in different cell layers at different points of pre- and postnatal development. Indeed, the level of apoptosis in the external granule cell layer gradually increases from week 26 until birth, whilst cells in the Purkinje layer only start to die postnatally [33, 34]. Such patterns of apoptosis can be modulated by extrinsic factors. For example, Purkinje cell apoptosis is enhanced* in utero* during fetal alcohol syndrome [35]. Since BK_Ca_ *α* channels are expressed in Purkinje neurones [36] and their activity is known to be augmented by ethanol [37], it seems likely that alcohol-evoked increases in BK_Ca_ *α* activity may contribute to the enhanced apoptosis observed in this neuronal cell layer.

Therefore, the data of this study show that the physical partnership of ANXA5 and BK_Ca_ *α* channels results in decreased Ca^2+^ sensitivity of the latter under conditions close to the physiological. The proposed mechanism may be particularly important for the programmed cell death mechanism, where membrane flippage removes the ANXA5 from the vicinity of the intracellular C-terminal of BK_Ca_ *α*, resulting in augmentation of K^+^ efflux and subsequent apoptosis.

## Figures and Tables

**Table 1:** Ca^2+^ concentration-response EC_50_ values for BK_Ca_ *α* channel of the control, ANXA5 siRNA knockdown, and ANXA5 overexpression models.

	Control (*n* = 8)		ANXA5 siRNA knockdown (*n* = 8)		ANXA5 overexpression (*n* = 8)	
	*I*/*I*_max_ at +60 mV	Va_50_ (mV)	*I*/*I*_max_ at +60 mV	Va_50_ (mV)	*I*/*I*_max_ at +60 mV	Va_50_ (mV)
EC_50_ [Ca^2+^] (*μ*M)	1.79 ± 0.57	2.07 ± 0.39	0.53 ± 0.09^*∗*^	0.62 ± 0.07^*∗*^	3.58 ± 0.48^*∗*^	4.42 ± 0.65^*∗*^

^*∗*^The values were considered as significantly different from the respective values of the control (*p* > 0.05).

**Table 2:** Normalized current (*I*/*I*_max_) at 60 mV and Va_50_ values for BK_Ca_ *α* channel of the control, ANXA5 siRNA knockdown, and ANXA5 overexpression models.

	Control (*n* = 8)		ANXA5 siRNA knockdown (*n* = 8)		ANXA5 overexpression (*n* = 8)	
	*I*/*I*_max_ at +60 mV	Va_50_ (mV)	*I*/*I*_max_ at +60 mV	Va_50_ (mV)	*I*/*I*_max_ at +60 mV	Va_50_ (mV)
[Ca^2+^] 300 nM	0.25 ± 0.07	74.5 ± 4.6	0.38 ± 0.07	67.5 ± 2.8	0.19 ± 0.05^*∗*^	79.5 ± 3.8^*∗*^
CORM-2 30 *μ*M	0.61 ± 0.1	55.9 ± 5.2	0.79 ± 0.06	44.1 ± 2.7	0.51 ± 0.06^*∗*^	57.5 ± 2.5^*∗*^
[Ca^2+^] 1 *µ*M	0.44 ± 0.08	60.0 ± 3.9	0.58 ± 0.10	54.8 ± 6.3	0.28 ± 0.03^*∗*^	73.2 ± 2.7^*∗*^

^*∗*^The values were considered as significantly different from the respective values of ANXA5 siRNA knockdown (*p* > 0.05).

## Abbreviations

ANXA5: Annexin A5
AVD: Apoptotic volume decrease
BK_Ca_*α*: Large conductance voltage-activated and calcium-sensitive potassium channel subunit
HEK 293 cells: Human embryonic kidney cells
HO-2: Heme oxygenase-2
TEA: Tetraethylammonium
FCCP: Carbonyl cyanide 4-(trifluoromethoxy)phenylhydrazone
HEPES: N-2-Hydroxyethylpiperazine-N′-2-ethanesulfonic acid
[Ca^2+^]_o_: Extracellular calcium concentration
[Ca^2+^]_i_: Intracellular calcium concentration
EGTA: Ethylene-glycol-tetra-acetic acid
CORM-2: Tricarbonyldichlororuthenium (II) dimer ([Ru(CO_3_)Cl_2_]_2_.
